# The Potential Relationship Between The Environmental Risk Factors And Social Cognition in Psychosis

**DOI:** 10.1192/j.eurpsy.2022.727

**Published:** 2022-09-01

**Authors:** S. Cicek, F. Karadag

**Affiliations:** 1 Cankiri State Hospital, Psychiatry, Cankiri, Turkey; 2 Gazi University Faculty of Medicine, Psychiatry, Ankara, Turkey

**Keywords:** social cognition, Psychosis, environmental risk factors

## Abstract

**Introduction:**

In schizophrenia research, little is known about the relationship of environmental exposures with social cognition deficits.

**Objectives:**

We aimed to investigate the relationship between social cognitive performance and well-defined environmental risk factors (childhood adversities, birth season, paternal age, obstetric complications, urban living i.e.) in schizophrenia.

**Methods:**

54 schizophrenia patients and 37 healthy controls (HCs) were included in our study. Participants in both groups were of similar age, gender, and educational level. Two theory of mind (ToM) tests (DEZIKÖ and RMET), and the Childhood Trauma Questionnaire (CTQ) were applied. ToM test scores among groups (patients with/ without risk factors, and HC) were compared using analysis of variance.

**Results:**

Overall, the schizophrenia group scored higher on the CTQ and performed worse on ToM tests than the HCs. Patients were more likely to report obstetric complications, advanced paternal age, winter and rural birth. Both the patients having high and low CTQ scores performed poorer on the RMET and false belief test than HCs. However, there was no significant difference in DEZİKÖ-total scores of patients with low CTQ scores and HCs. Patients with advanced paternal age at birth achieved lower faux pas sub-scores. Urban birth and RMET scores were positively correlated in patients.

**Conclusions:**

Our findings suggest the environmental factors such as childhood traumas, advanced paternal age, and rural birth seem to negatively affect the social cognitive performance of schizophrenia patients.

**Disclosure:**

No significant relationships.

